# Effectiveness of deep learning classifiers in histopathological diagnosis of oral squamous cell carcinoma by pathologists

**DOI:** 10.1038/s41598-023-38343-y

**Published:** 2023-07-19

**Authors:** Shintaro Sukegawa, Sawako Ono, Futa Tanaka, Yuta Inoue, Takeshi Hara, Kazumasa Yoshii, Keisuke Nakano, Kiyofumi Takabatake, Hotaka Kawai, Shimada Katsumitsu, Fumi Nakai, Yasuhiro Nakai, Ryo Miyazaki, Satoshi Murakami, Hitoshi Nagatsuka, Minoru Miyake

**Affiliations:** 1 Department of Oral and Maxillofacial Surgery, Kagawa University Faculty of Medicine, 1750-1 Ikenobe, Miki, Kagawa 761-0793 Japan; 2 Department of Oral and Maxillofacial Surgery, Kagawa Prefectural Central Hospital, 1-2-1, Asahi-Machi, Takamatsu, Kagawa 760-8557 Japan; 3 Department of Oral Pathology and Medicine, Graduate School of Medicine, Dentistry and Pharmaceutical Sciences, Okayama University, Okayama, 700-8558 Japan; 4 Department of Pathology, Kagawa Prefectural Central Hospital, 1-2-1, Asahi-Machi, Takamatsu, Kagawa 760-8557 Japan; 5 Department of Electrical, Electronic and Computer Engineering, Faculty of Engineering, Gifu University, 1-1 Yanagido, Gifu, Gifu 501-1193 Japan; 6 Center for Healthcare Information Technology, Tokai National Higher Education and Research System, 1-1 Yanagido, Gifu, Gifu 501-1193 Japan; 7 Department of Oral Pathology, Graduate School of Oral Medicine, Matsumoto Dental University, 1780 Hirooka-Gobara, Shiojiri, Nagano 399-0781 Japan

**Keywords:** Oral cancer, Oral pathology, Pathology

## Abstract

The study aims to identify histological classifiers from histopathological images of oral squamous cell carcinoma using convolutional neural network (CNN) deep learning models and shows how the results can improve diagnosis. Histopathological samples of oral squamous cell carcinoma were prepared by oral pathologists. Images were divided into tiles on a virtual slide, and labels (squamous cell carcinoma, normal, and others) were applied. VGG16 and ResNet50 with the optimizers stochastic gradient descent with momentum and spectral angle mapper (SAM) were used, with and without a learning rate scheduler. The conditions for achieving good CNN performances were identified by examining performance metrics. We used ROCAUC to statistically evaluate diagnostic performance improvement of six oral pathologists using the results from the selected CNN model for assisted diagnosis. VGG16 with SAM showed the best performance, with accuracy = 0.8622 and AUC = 0.9602. The diagnostic performances of the oral pathologists statistically significantly improved when the diagnostic results of the deep learning model were used as supplementary diagnoses (p-value = 0.031). By considering the learning results of deep learning model classifiers, the diagnostic accuracy of pathologists can be improved. This study contributes to the application of highly reliable deep learning models for oral pathological diagnosis.

## Introduction

Oral cancer is one of the most common malignancies in both developing and developed countries^1^. Squamous cell carcinoma (SCC) represents the majority of the histopathological types of oral cancer. Oral cancer includes cancer of the lips and other cancers that begin from the parts of the oral cavity. It is the 16th most common malignant tumor in the world and the 15th most common cause of death^2^. For every 100,000 people worldwide, there are four incidents of oral cancer^3^. Therefore, the importance and workload of pathologists in diagnosing this disease are increasing. Pathologists must make many histological diagnoses, and large amounts of experience and learning are required to achieve an accurate diagnosis.

The success of deep learning strategies using convolutional neural networks (CNNs) for images in the non-medical domain has tremendously influenced the analysis of medical images. In recent years, these deep learning algorithms have been used for image classification in various medical fields^4,5^. Studies involving these deep learning techniques have not only applied them to radiographic images via X-ray images^6^ and computed tomography (CT) data^7^ but also involved clinical studies using histopathological images^8^.

Although the classification performances of deep learning models have greatly improved over time, they alone cannot be used to obtain completely accurate classification diagnoses. Similarly, pathologists cannot always make correct diagnoses. A histopathological diagnosis is an informed opinion made by a pathologist using a subjective assessment of morphological features. In these diagnoses, gray areas are inevitably encountered that vary widely among observers. This variation can occur because of variable cutoff values in the morphological continuum or variable weights given to different morphological features^9^. Therefore, double-checking is a useful technique in histopathological diagnoses and has been adopted in clinical practice. As a form of double-checking, we hypothesize that the use of deep learning may contribute to improving the accuracy of histopathological diagnoses.

The primary purpose of this study is to identify an effective histological classifier from histopathological images of oral squamous cell carcinoma using a deep learning CNN model and then to clarify the classification of the performance of the classifier. The second purpose is to show whether the learning results of the identified effective deep learning classifier model can contribute to improving the diagnostic performance of oral pathologists.

## Results

### Performance comparison of different CNN models

Table 1 shows the results of the performance metrics obtained with and without a learning rate scheduler for the SGDM and SAM optimizers on VGG16 and ReaNet50. With the introduction of learning rate scheduling, SGDM exhibited improved performance metrics except for the area under the curve (AUC). Comparing SAM and SGDM, VGG16 had higher performance metrics under all conditions, and ResNet50 had higher performance metrics for all conditions except for AUC when SAM was used. Of all model combinations, VGG16 with SAM showed the highest performance. In this study, the best deep learning model was found to be VGG16 with SAM as the optimizer.

**Table 1:** Performance comparison of each CNN model.

CNN model	Optimizer	Learning rate	Accuracy	Precision	Recall	F1 score	AUC
			SD	SD	SD	SD	SD
			95% CI	95% CI	95% CI	95% CI	95% CI
VGG16	SGDM	Without scheduler	0.8522	0.8125	0.8584	0.8301	0.9601
			0.0051	0.0030	0.0028	0.0024	0.0008
			0.850–0.854	0.811–0.813	0.857–0.859	0.829–0.831	0.960–0.960
		With scheduler	0.8575	0.8255	0.8551	0.8384	0.9520
			0.0022	0.0033	0.0033	0.0025	0.0019
			0.857–0.858	0.824–0.827	0.854–0.856	0.839–0.858	0.951–0.953
	SAM	With scheduler	0.8622	0.8319	0.8589	0.8438	0.9602
			0.0020	0.0030	0.0028	0.0024	0.0008
			0.862–0.863	0.831–0.833	0.858–0.860	0.843–0.845	0.960–0.961
ResNet50	SGDM	Without scheduler	0.8388	0.7932	0.8491	0.8152	0.9510
			0.0024	0.0033	0.0038	0.0027	0.0021
			0.838–0.840	0.792–0.794	0.848–0.851	0.814–0.816	0.950–0.952
		With scheduler	0.8440	0.8017	0.8489	0.8218	0.9483
			0.0096	0.0147	0.0225	0.0179	0.0125
			0.841–0.847	0.797–0.807	0.841–0.857	0.815–0.828	0.944–0.953
	SAM	With scheduler	0.8457	0.8038	0.8492	0.8232	0.9507
			0.0018	0.0033	0.0038	0.0027	0.0021
			0.845–0.846	0.803–0.805	0.848–0.851	0.822–0.824	0.950–0.951

*SD* standard deviation, *CI* confidence interval, *AUC* area under the ROC curve.

### Comparison of oral pathologists' diagnoses with and without deep learning assistance

Table 2 shows the AUC, macro-mean, and micro-mean values for each class, including normal, SCC, and others for each oral pathologist. Furthermore, the highest AUC without an assistive diagnosis was for oral pathologist #4, who obtained a macro average of 0.95 (95% confidence interval; 0.942–0.950) and a micro average of 0.95 (95% confidence interval; 0.946–0.955). Considering the diagnosis, the macro average was 0.98 (95% confidence interval; 0.976–0.980), and the micro average was 0.95 (95% confidence interval; 0.976–0.982).

**Table 2:** Comparison of the oral pathologists' diagnoses with and without deep-learning-assisted diagnoses.

Evaluator	Normal [95% CI]	SCC [95% CI]	Others [95% CI]	Macro [95% CI]	Micro [95% CI]
Oral pathologist #1					
With assistive diagnosis	0.97 [0.968–0.979]	0.99 [0.984–0.989]	0.94 [0.929–0.942]	0.97 [0.960–0.966]	0.97 [0.958–0.965]
w/o assistive diagnosis	0.75 [0.732–0.759]	0.88 [0.871–0.882]	0.79 [0.784–0.804]	0.80 [0.795–0.810]	0.79 [0.776–0.791]
Oral pathologist #2					
With assistive diagnosis	0.98 [0.978–0.986]	0.94 [0.936–0.947]	0.88 [0.870–0.885]	0.93 [0.929–0.939]	0.93 [0.927–0.937]
w/o assistive diagnosis	0.91 [0.902–0.922]	0.85 [0.847–0.863]	0.82 [0.811–0.828]	0.86 [0.854–0.867]	0.83 [0.819–0.833]
Oral pathologist #3					
With assistive diagnosis	0.73 [0.714–0.741]	0.91 [0.896–0.909]	0.83 [0.823–0.839]	0.82 [0.806–0.818]	0.88 [0.879–0.889]
w/o assistive diagnosis	0.56 [0.549–0.572]	0.82 [0.816–0.832]	0.71 [0.712–0.732]	0.70 [0.691–0.704]	0.81 [0.798–0.812]
Oral pathologist #4					
With assistive diagnosis	1.00 [0.998–0.999]	0.98 [0.979–0.984]	0.96 [0.950–0.959]	0.98 [0.976–0.980]	0.98 [0.976–0.982]
w/o assistive diagnosis	0.97 [0.968–0.977]	0.97 [0.965–0.972]	0.90 [0.893–0.905]	0.95 [0.942–0.950]	0.95 [0.946–0.955]
Oral pathologist #5					
With assistive diagnosis	0.96 [0.948–0.964]	0.95 [0.945–0.953]	0.92 [0.917–0.928]	0.94 [0.941–0.949]	0.96 [0.952–0.959]
w/o assistive diagnosis	0.92 [0.902–0.922]	0.89 [0.884–0.900]	0.86 [0.845–0.860]	0.89 [0.884–0.897]	0.91 [0.900–0.913]
Oral pathologist #6					
With assistive diagnosis	1.00 [0.996–0.997]	0.98 [0.979–0.968]	0.97 [0.963–0.968]	0.98 [0.982–0.985]	0.98 [0.980–0.983]
w/o assistive diagnosis	0.94 [0.928–0.943]	0.85 [0.837–0.852]	0.75 [0.749–0.768]	0.85 [0.843–0.854]	0.85 [0.836–0.849]

*95% CI* 95% confidence interval.

Oral pathologist #1 was most effective when an assistive diagnosis was used. A macro mean of 0.80 (95% confidence interval; 0.795–0.810) and a micro mean of 0.79 (95% confidence interval; 0.776–0.791) were obtained without an assistive diagnosis. When the assistive diagnosis component was used, the macro average was 0.97 (95% confidence interval; 0.960–0.966), and the micro average was 0.97 (95% confidence interval; 0.958–0.965).

The diagnostic performances of all pathologists were improved in terms of the AUC using the assistive diagnosis technique.

**Figure 1:**
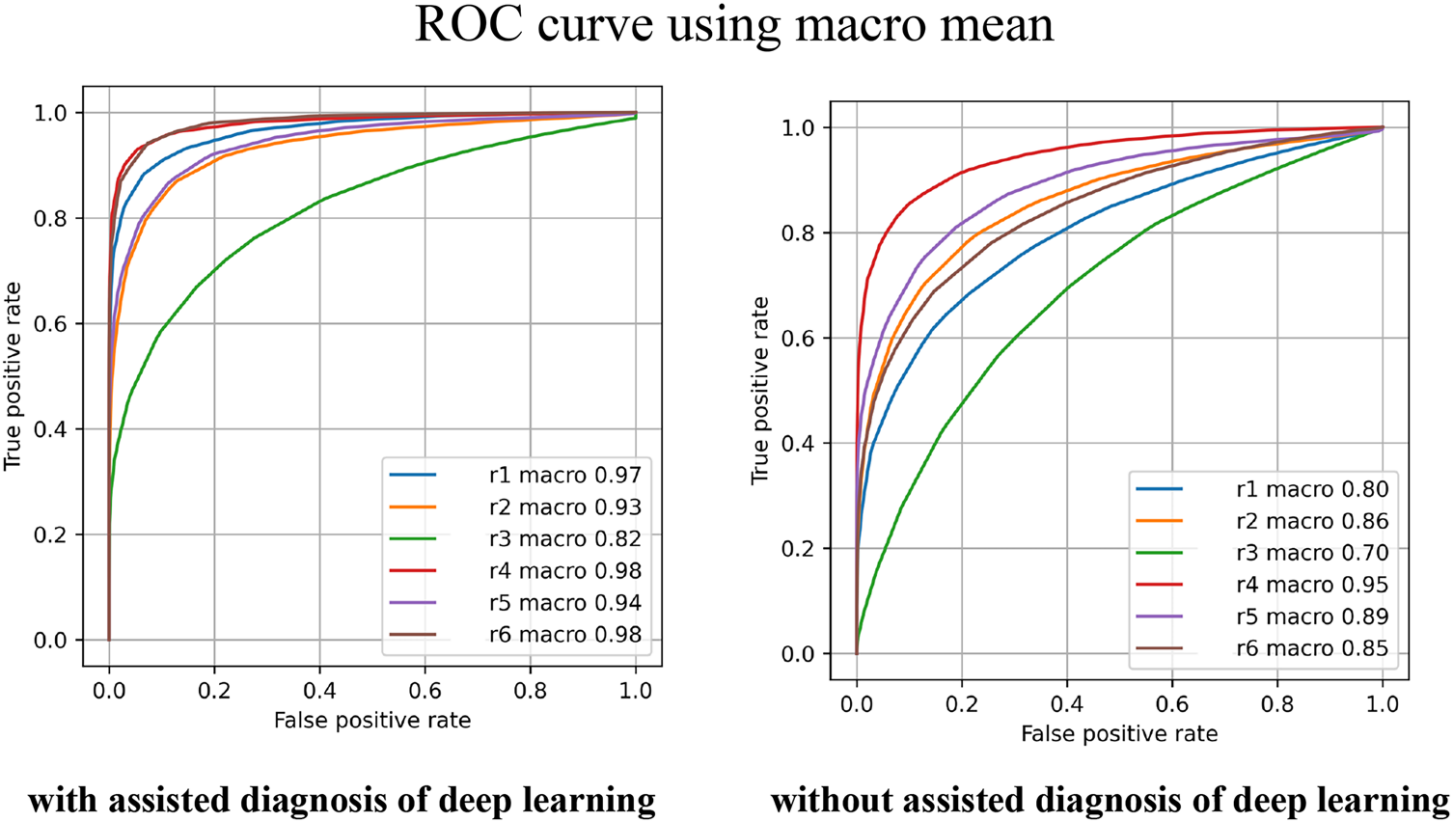
Comparison of oral pathologists' diagnoses with and without deep learning assistance considering the ROC curve using macro mean values.

**Figure 2:**
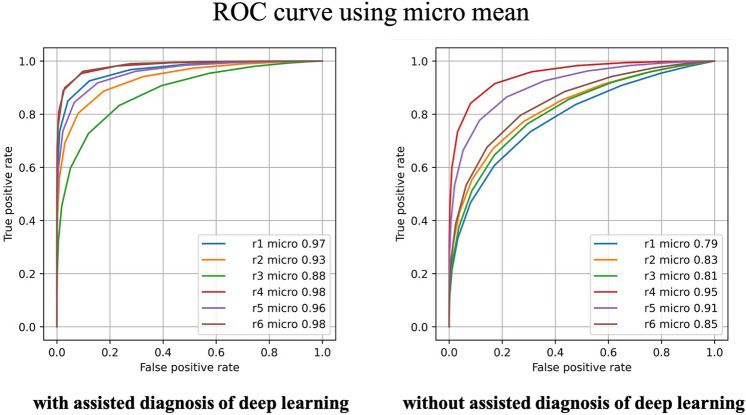
Comparison of oral pathologists' diagnoses with and without deep learning assistance considering the ROC curve using micro mean values.

The receiver operating characteristic curve (ROC) curves of the macro and micro averages with and without the use of assistive diagnosis are shown in Figs. 1 and 2. Both the macro- and micro-means show an improvement in terms of the AUC for both the examined oral pathologists.

### Statistical comparison of oral pathologist's diagnoses with and without deep learning assistance

**Figure 3:**
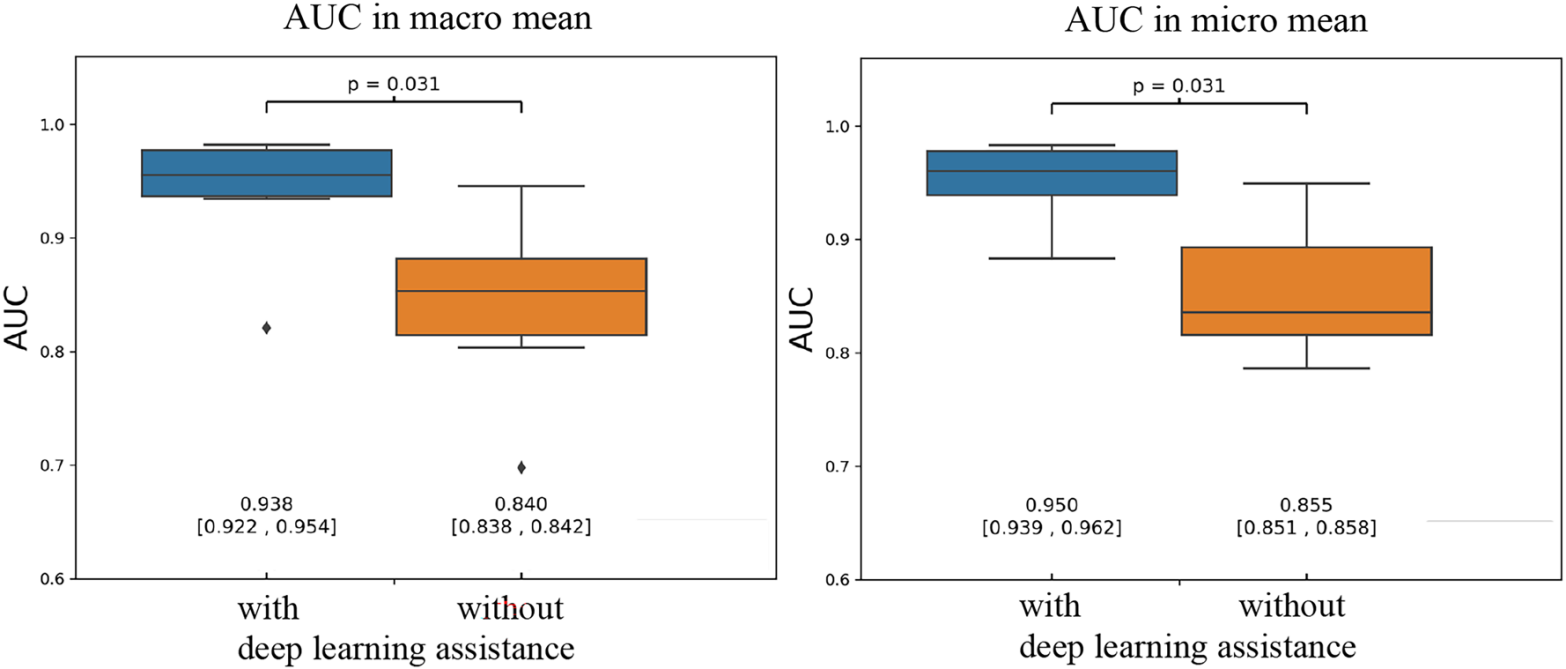
Statistical comparison of the oral pathologist's diagnoses with and without deep learning assistance.

Figure 3 shows the statistical evaluation results obtained with and without deep learning assistance in terms of the macro- and micro-AUC mean values. A statistically significant difference was observed between the macro and micro mean values (p value = 0.031 for both the macro and micro mean). In addition, the effect size of the deep-learning-assisted diagnosis for improving the diagnostic performance of the oral pathologists was 1.46 for the macro average and 2.04 for the micro average, which correspond to “huge” and “very large” effects, respectively. Please refer to Appendix S2 for a further explanation of the effect size.

## Discussion

This study demonstrated that the most effective classification model for classifying histopathological images of oral squamous cell carcinoma using deep learning uses VGG16 with a learning rate scheduler and the SAM optimizer. Diagnoses using deep-learning assistance were shown to contribute to the improvement of the diagnostic accuracy of oral pathologists by considering the learning results of the classifier that were obtained using the best model.

This study first identified an optimized CNN model for the considered dataset. The best model used the SAM optimizer with VGG16 and a learning rate scheduler, as mentioned previously. The SAM optimizer has been recently reported as a deep learning optimization method that performed well for publicly available datasets^10^ and classifiers using medical images^11,12^. Similar results were obtained using other deep learning classifiers researched herein. Although they did not perform as well as SAM, in each CNN model using SGDM as an optimizer, the introduction of a learning rate scheduler was effective in improving the performance within a limited number of epochs. Comparing the VGG16 and ResNet50 CNN models, the VGG16 performed better on the present dataset and hyperparameters. The VGG16 is a CNN architecture that has been demonstrated to improve robustness depending on the model environment^13^, and this was also observed in this study.

In recent years, studies have used classifiers based on deep learning techniques that are applied to pathological tissue images of the head and neck region. Various methods have been used for verification, and the images that are used vary depending on public and facility-specific data^14^, which makes the cross-sectional comparisons of classification accuracy difficult. Previous studies using CNN classifiers for the histopathological diagnosis of oral squamous cell carcinoma have reported accuracies of 77.9% to 90.1%^14–16^. Most studies have divided oral squamous cell carcinoma into normal tissue or benign and malignant tumors. In this study, three other categories were used, including normal, oral squamous cell carcinoma, and inflammatory response. Additionally, we targeted all cropped images that contained cells. Many factors make diagnosis difficult. Despite such complex conditions, the proposed CNN model achieved a high classification diagnostic performance for the multiclass classification of complex datasets.

We analyzed the effectiveness of deep-learning-assisted diagnosis using ROC curves and AUC data when used to aid oral pathologists. In this study, we considered both macro and micro averaging. The macro average values can reflect all classes similarly, whereas the micro average can reflect the bias considering the amount of data in each class. In this study, both the macro- and micro-average AUC evaluations showed statistically significant differences. Therefore, the use of deep-learning-assisted diagnosis was shown to contribute greatly to improving the diagnostic performances of oral pathologists. A previous study reported that the supplementary use of the results of artificial intelligence resulted in improved diagnostic accuracy. Other techniques, including plain X-ray imaging^17^, ultrasonography^18^, and histopathological diagnosis for breast lesions^19^, have provided both correct and incorrect evaluations. Conversely, in this study, we evaluated macro- and micro-averaged AUC techniques using continuous confidence, and this is the first study to evaluate the effectiveness of deep-learning-assisted diagnoses in oral histopathology. Therefore, this study is of great significance.

Each image segmented from the WSI image was classified into three. In general, pathologists use a single specimen slide to make an overall diagnosis, and they consider the condition of the surrounding tissue before making a final decision. Therefore, making confident diagnostic decisions from only one segmented image is challenging. In this study, we posited that the use of deep-learning-assisted diagnosis positively affects the confidence of pathologists. Importantly, we statistically demonstrated the effectiveness of deep learning diagnostic aids. This is the first study to demonstrate the improved diagnostic performances of pathologists using ROCAUC evaluation methods. In addition, we also demonstrated the effect size related to the auxiliary diagnosis provided by deep learning^20^. Effect sizes may be used to determine the number of observers that will be present in future similar studies. The results of this study may provide a basis for the application of reliable deep learning methods in histopathological diagnoses.

This study has several limitations. First, only a few CNN models were verified, and many other optimizers and learning rate schedulers were not investigated. To verify the use of more complex CNN models, sufficient resources that can withstand the required computational costs are needed. Second, the pathological tissue images were verified at only one facility, and the verification of external validity using external data is also required to confirm the effectiveness of more robust auxiliary deep learning diagnosis methods. Third, dataset-splitting techniques can affect the generalizability of deep learning techniques. In this study, we subdivided five sample specimens, extracted 7918 images for deep learning, and divided the training data into test data from those images. Considering the similarity of the data, comparing the evaluation methods for the learning and test data for each histopathological specimen will be required in future studies. Fourth, to evaluate the effectiveness of deep learning assistance, we first made a diagnosis without using deep learning and then made a diagnosis using deep learning assistance. The interval between evaluations varied according to the pathologist who performed each evaluation. The same test sample may affect the pathologist's subjective judgment; therefore, considering evaluations after a long period, such as two weeks, is necessary.

## Conclusions

In this study, we identified an effective histological classifier from histopathological images of oral squamous cell carcinoma and clarified the classification performance of this classifier using deep learning. The most effective model was VGG16, with a learning rate scheduler and SAM optimizer. This system was statistically demonstrated to improve the diagnostic accuracy of pathologists by referring to the learning results of the classifiers that have undergone deep learning. This study provides a basis for applying reliable deep learning systems in the field of oral pathology diagnosis.

## Materials and methods

### Study objectives

**Figure 4:**
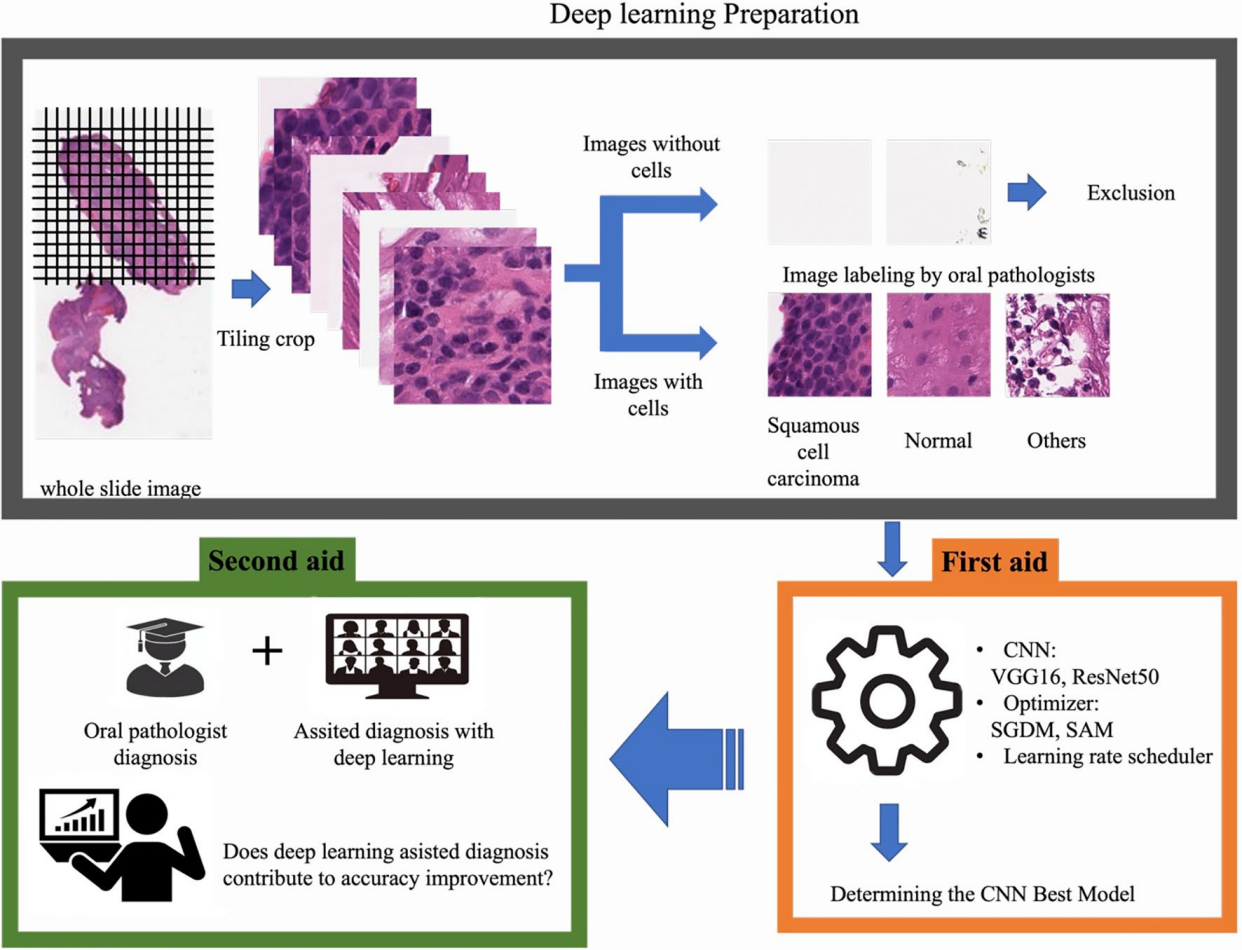
Overall flow of the research on deep learning classification models for oral histopathology.

The first objective of this study is to identify an effective histological classifier from histopathological images of oral squamous cell carcinoma using supervised learning and a deep learning CNN model, as well as to clarify its classification performance. The second objective is to evaluate whether it can contribute to the diagnostic performance of a pathologist when referring to the learning results of the identified optimal deep learning model. A schematic of this study is shown in Fig. 4.

### Ethics statement

This study was approved by the Institutional Review Board (IRB) of the Kagawa Prefectural Central Hospital Ethics Committee (the Institutional Review Boards of Kagawa Prefectural Central Hospital, approval number: 1071). The IRB reviewed our study, which is a non-interventional retrospective study design. It is an analytical study with fully anonymized data, and the need for informed consent was waived. Because the data were evaluated retrospectively, pseudonymously, and were solely obtained for treatment purposes, a requirement of informed consent was waived by the IRB of the Kagawa Prefectural Central Hospital Ethics Committee. Therefore, written and verbal informed consent was not obtained from the patients from whom pathological specimens were obtained. This research uses existing sample information, and obtaining direct informed consent from all research subjects is difficult. In addition, at the request of research subjects or their representatives directly to the hospital ethics committee, informed consent was denied by timed opportunities to refuse participation when requested to use specimen information that could identify research subjects or to provide it to other research institutions. This study was conducted in accordance with the Declaration of Helsinki and according to the rules and protocol approved by the IRB.

### Image data preparation

The dataset used slide glasses of five biopsy specimens stained with hematoxylin and eosin (H & E). The five specimens were three cases of tongue cancer and two cases of oral floor cancer [four cases for men, one case for women; average age: 73 years (47 to 90 years)].

The glass slides were scanned with an Aperio AT2 scanner (Leica Biosystems, Buffalo Grove, Illinois) at 40-times magnification to create a whole slide image (WSI). The created WSI was tiled using OpenSlide (version 3.4.1, University of Pittsburgh, Pittsburgh, PA) to create small cropped images. The cropped images were output in portable network graphics (PNG) format at 256-by-256 pixels.

### Image data annotation and selection

Each manually cropped and created image was labeled by two oral pathologists for each manually cropped image. They labeled each image independently. The images were labeled according to the consistency of the diagnosis of the two pathologists; the disagreed-upon images required an additional diagnosis by a highly specialized physician and were decided by a majority vote. In addition, all images that did not contain cells were excluded from analysis in this study. The labeling methods were defined using the following three categories: normal and SCC were classified according to Nandini’s nuclear grading system^21^. These labels include (1) normal cells, including cells with an oval nuclear shape, round nuclear shape, regular nuclear membrane, no chromatin clumps, and abnormal mitotic figures inconspicuous nucleoli; (2) squamous cell carcinoma, including cells with an irregular nuclear shape, irregular nuclear membrane, some chromatin clumps, abnormal mitotic figures, and distinct nucleoli; and (3) others, which included reactive, hyperplastic histology, inflammatory images, necrotic tissue or tissue fragments, cells or tissues other than epithelium, atypical but atypical or weak for cancer, or atypical of unknown significance. A total of 7918 images (989 normal, 1167 squamous cell carcinoma, and 5762 other) were professionally labeled.

### Selection of CNN model architecture

We selected two well-known CNN models, VGG16^22^ and ResNet50^23^. VGG16 is a CNN model developed by a research group at Oxford University in 2014, and it is a high-precision model that was placed second in the Imagenet image recognition competition. ResNet is a CNN model that can solve the vanishing gradient problem that results in learning difficulties when the CNN structure is multilayered, achieved by incorporating shortcut connections; furthermore, it can achieve a high prediction accuracy^23^. We selected ResNet50, which is a CNN with a depth of 50 layers.

### Data augmentation

A data augmentation method was used to increase the number of images in the training dataset. This allows the improvement of the efficiency of a model, overcomes the problem of overfitting, and makes the model more generalized^24^. In this study, rotation (− 18° to 18° range), flip (horizontal and vertical), and conversion (30% up/down/left/right) were performed randomly, and the missing part of the image was complemented using the reflection method.

### Dataset and model training

The CNN model training was generalized using K-fold cross-validation in the deep learning algorithm. Model validation was evaluated using a four-fold cross-validation technique to avoid overfitting and bias and minimize the generalization error. The dataset was divided into four random subsets using stratified sampling, and the same class distribution was maintained for training, validation, and testing across all subsets^25^. Within each fold, the dataset was split into separate training and testing datasets at a ratio of 90:10. Additionally, the validation data consisted of 10% of the training data. The model performance evaluation used the average of the analysis results for each fold to obtain the results for the entire dataset.

For the loss function, the cross-entropy obtained from the following equation was used:

$$Cross-entropy\,Loss= -\sum_{i=0}^{n-1}{t}_{i}\,{log}_{e}{y}_{i} .$$

t_i_ is the true label; y_i_ is the predicted probability of class i.

### Optimizer selection

We chose stochastic gradient descent with momentum (SGDM) and sharpness aware minimization (SAM) as the optimization algorithms for this study.

The stochastic gradient descent method is a commonly used algorithm, and we selected SDGM, which is given momentum to suppress vibrations when considering the moving average^26^. In this study, the momentum was set to 0.9. SGDM is expressed by the following formula:

$$\Delta {w}_{t}=\mathrm{\alpha }\Delta {w}_{t-1}- \eta \nabla \mathrm{L}\left(\mathrm{w}\right) ,$$

$${w}_{t}={w}_{t-1}+\Delta {w}_{t} .$$

w_t_ is the parameter; η is the learning rate; ∇L (w) is the differentiation with parameters of the loss function; α is the momentum.

SAM is a learning algorithm that targets parameters with a minimal loss and flat surroundings^10^. We selected SAM because it is a learning algorithm that demonstrates high prediction accuracy and enhanced robustness. The loss function of SAM is defined by Eq. (1). SAM is minimized using Eq. (2), which includes the loss function. The neighborhood size of SAM was selected by referring to the optimal neighborhood size of 0.025 when the number of epochs was 300, according to previous research^11^.

1 $$\underset{w}{\mathrm{min}}{L}_{S}^{SAM}\left(w\right)+\lambda {\Vert w\Vert }_{2}^{2}$$

2 $${L}_{S}^{SAM}\left(w\right)=\underset{{\Vert \varepsilon \Vert }_{p}\le \rho }{\mathrm{max}}{L}_{s}(w+\varepsilon )$$

S is the set of data; w is the parameter; λ is the L2 regularization coefficient; L_s_ is the loss function; ρ is the neighborhood size.

### Deep learning procedure

#### Learning rate scheduler

Learning rate decay is a method used to improve the learning efficiency and generalization performance of deep learning models, and it is a method that lowers the learning rate as learning progresses^23^. The learning rate decay used in this study can be defined by the following equation, with an initial learning rate of 0.01:

$${lr}_{new}=\frac{{lr}_{current}}{(1+decay\,rate\times epoch)}.$$

#### Deep learning analysis procedure

All deep learning analyses were performed using a 64 bit Ubuntu 18.04.5 LTS operating system (Canonical Ltd., London, UK) and NVIDIA GeForce Tesla V100-SXM2 16 GB graphics processing unit (NVIDIA, Sta. Clara, CA, USA). The process of deep learning classification was implemented using Keras (version.2.7.0).

All CNN models were trained at 300 epochs and 32 mini-batch sizes and did not use premature termination. These deep learning analysis processes were repeated 30 times for each model, and different random seeds were used for each model.

### Performance metrics

All deep learning models were evaluated in terms of their accuracy, precision, recall, specificity, F1 score, and AUC calculated from ROC as performance metrics. More information on each performance metric can be found in Appendix S1.

### Comparison of the diagnostic performances of oral pathologists with and without a deep-learning-assisted diagnosis

#### Composition of oral pathologists

Six oral pathologists participated in this study—three board-certified specialists in oral pathology and three specialists in oral pathology who have not yet been board-certified.

#### Evaluation method using ROCAUC

Each oral pathologist was informed about the composition of the images (normal and squamous cell carcinoma), and they reviewed the images individually to make diagnoses. No time limit was provided for diagnosis. First, diagnoses were made without the deep learning assistance, and later deep learning assistance was used in the diagnoses. The correct diagnosis for each image was not communicated to the oral pathologist evaluators until after the two tests were completed. The pathologists performed the tests individually and promised not to share their results with the other observers. The diagnostic method used was the continuous confidence method, in which scores were given on a free scale according to various criteria. The method used a visual scale from 0 to 100 to determine the certainty of normal, squamous cell carcinoma, and other diagnoses for each test image. Using the results, the SoftMax function was used to convert the total output values of the three categories to 1.0 (100%).

In this study, we analyzed the effectiveness of deep-learning-assisted diagnosis using the ROC curve and ROCAUC for aiding the diagnoses of oral pathologists. Using macro- and micro-average values of the results, we compared the effect of deep-learning-assisted diagnosis using ROC and evaluated the effect of using deep learning on the diagnostic performances of oral pathologists.

### Statistical analysis

A statistical assessment of the classification performance of each CNN model was performed for the results that were obtained over the course of 30 analyses. All performance metrics used in this study were statistically analyzed using the JMP Statistical Software Package Version 14.2.0 for Macintosh (SAS Institute Inc., Cary, NC, USA). A P-value of less than 0.05 was considered statistically significant. The normal distribution of continuous variables was evaluated using the Shapiro–Wilk test. The difference in classification performance between each CNN model was calculated for each metric using the Wilcoxon signed-rank test. The effect size^27^ was calculated as Hedges' g. More information on the effect size can be found in Appendix S2.

The effect size is a metric that was proposed by Cohen that is determined based on the criteria proposed by Sawilloski^28^. A huge effect is defined as 2.0 or more, a very large effect is 1.0, a large effect is 0.8, a medium effect is 0.5, a small effect is 0.2, and a very small effect is 0.01 or less.

## Supplementary Information

Supplementary Information.

## Data Availability

The datasets used and/or analyzed during the current study are available from the corresponding author upon reasonable request.
