# Supplementary material for: Effectiveness of deep learning classifiers in histopathological diagnosis of oral squamous cell carcinoma by pathologists
Source: Sci Rep. 2023 Jul 19;13:11676. doi: 10.1038/s41598-023-38343-y (PMC10356919; doi:10.1038/s41598-023-38343-y)
Supplement: Supplementary file 1 — Supplementary Information. [file 41598_2023_38343_MOESM1_ESM.docx]

Effectiveness of Deep Learning Classifiers in Histopathological Diagnosis of Oral Squamous Cell Carcinoma by Pathologists

Shintaro Sukegawa ^1,2,3^ *, Sawako Ono ^4^, Futa Tanaka ^5^, Yuta Inoue ^5^, Takeshi Hara ^5,6^, Kazumasa Yoshii ^5^, Keisuke Nakano ^3^, Kiyofumi Takabatake ^3^, Hotaka Kawai ^3^, Shimada Katsumitsu ^7^, Fumi Nakai ^1^, Yasuhiro Nakai ^1^, Ryo Miyazaki ^1^, Satoshi Murakami ^7^, Hitoshi Nagatsuka ^3^, Minoru Miyake ^1^

^1^ Department of Oral and Maxillofacial Surgery, Kagawa University School of Medicine, 1750-1 Ikenobe, Miki, Kagawa 761-0793, Japan

^2^ Department of Oral and Maxillofacial Surgery, Kagawa Prefectural Central Hospital, 1-2-1, Asahi-machi, Takamatsu, Kagawa 760-8557, Japan

^3^ Department of Oral Pathology and Medicine, Graduate School of Medicine, Dentistry and Pharmaceutical Sciences, Okayama University, Okayama 700-8558, Japan

^4^ Department of Pathology, Kagawa Prefectural Central Hospital, 1-2-1, Asahi-machi, Takamatsu, Kagawa 760-8557, Japan

^5^ Department of Electrical, Electronic and Computer Engineering, Faculty of Engineering, Gifu University, 1-1 Yanagido, Gifu, Gifu 501-1193, Japan

^6^ Center for Healthcare Information Technology, Tokai National Higher Education and Research System, 1-1 Yanagido, Gifu, Gifu 501-1193 Japan

^7^ Department of Oral Pathology, Graduate School of Oral Medicine, Matsumoto Dental University, 1780 Hirooka-Gobara, Shiojiri, Nagano, 399-0781, Japan.

**Appendix.S1**

***Performance metrics***

We evaluated the performance metrics, with accuracy, precision, recall, and the F1 score as defined in Equations 3–7, along with the receiver operating characteristic curve (ROC), and the area under the ROC curve (AUC). The ROC curves were shown for the complete dataset from the 4-fold cross-validation, producing the median AUC value.

In the following equations, TP, TN, FP, and FN represent true positive (normal correctly identified), true negative (abnormal correctly identified), false positive (abnormal incorrectly identified), and false negative (normal incorrectly identified) results, respectively.

$accuracy=\frac{TP + TN}{TP + FP + TN + FN}$ (1)

$precision=\frac{\mathrm{TP}}{TP + FP}$ (2)

$recall=\frac{\mathrm{TP}}{TP + FN}$ (3)

$F1 score=2\times\frac{precision \times recall}{precision + recall}$ (4)

**Appendix.S2**

***Effect size***

Effect size is a measure of the strength of the relationship between two variables in a population and is a sample-based estimate of that amount. In this study, effect sizes were used to evaluate the effect of deep learning on the diagnostic performance of pathologists. The effect size was calculated using Hedges' g with the following formula.

$$Hedges' g=\frac{{|M}_{1}-M_{2}|}{s}$$

$$s=\sqrt{\frac{{(n}_{1}-1)s_{1}^{2}+(n_{2}-1)s_{2}^{2}}{n_{1}+n_{2}-2}}$$

Impact of deep learning on pathologist diagnostic performance:

M1 and M2 are the means for the pathologist diagnostic performance(with/without assistive diagnosis of deep learning) and pathologist diagnostic performance (with/without assistive diagnosis of deep learning), respectively. s1 and s2 are the standard deviations for the pathologist diagnostic performance(with/without assistive diagnosis of deep learning) and pathologist diagnostic performance(with/without assistive diagnosis of deep learning), respectively. n1 and n2 are the numbers for the pathologist diagnostic performance(with/without assistive diagnosis of deep learning) and pathologist diagnostic performance (with/without assistive diagnosis of deep learning), respectively.
